# Persistent joint pain and arm function in former baseball players

**DOI:** 10.1016/j.jseint.2021.05.001

**Published:** 2021-06-29

**Authors:** Garrett S. Bullock, Kristen F. Nicholson, Brian R. Waterman, Eric Niesen, Paul Salamh, Charles A. Thigpen, Ellen Shanley, Laurie L. Devaney, John M. Tokish, Gary S. Collins, Nigel K. Arden, Stephanie R. Filbay

**Affiliations:** a Centre for Sport, Exercise and Osteoarthritis Research Versus Arthritis, University of Oxford, Oxford, UK; b Nuffield Department of Orthopaedics, Rheumatology, and Musculoskeletal Sciences, University of Oxford, Oxford, UK; c Department of Orthopaedic Surgery, Wake Forest School of Medicine, Winston-Salem, NC, USA; d Athletic Department, University of Georgetown, Washington DC, USA; e Krannert School of Physical Therapy, University of Indianapolis, Indianapolis, IN, USA; f ATI Physical Therapy, Greenville, SC, USA; g Department of Kinesiology, College of Agriculture, Health, and Natural Resources, University of Connecticut, Storrs, CT, USA; h Orthopedics & Sports Medicine, Mayo Clinic, Phoenix, AZ, USA; i Centre for Statistics in Medicine, Nuffield Department of Orthopaedics, Rheumatology, and Musculoskeletal Sciences, University of Oxford, Oxford, UK; j Oxford University Hospitals NHS Foundation Trust, Oxford, UK; k Centre for Health, Exercise and Sports Medicine, Faculty of Medicine, Dentistry and Health Sciences, University of Melbourne, Melbourne, VIC, Australia

**Keywords:** Shoulder, Elbow, SANE, Osteoarthritis, Professional, College, Pitching

## Abstract

**Background:**

Baseball has specific sport and positional demands that may modify joint pain compared with other sports. Persistent joint pain reduces function and is an underlying reason for seeking medical care. The pain and functional status of players after they stop competitive play are unknown. Such knowledge can assist clinicians in creating personalized physical examinations and interventions for baseball players as they transition to retirement. The purpose of this study was to (1) evaluate persistent joint pain and arm function in former baseball players and (2) determine whether playing position is associated with increased odds of joint pain and reduced arm function in former baseball players.

**Methods:**

A cross-sectional survey was performed. Eligibility criteria consisted of (1) played ≥1 collegiate baseball season, (2) aged ≥18 years, and (3) formerly played baseball (currently retired). Outcomes assessed included persistent joint pain and Single Assessment Numeric Evaluation (SANE). Explanatory variables included playing position (position, two-way, or pitcher). Multivariable logistic and linear regressions were performed. Models were adjusted for age, body mass index, arm dominance, playing standard, years played baseball, and injury and surgery history.

**Results:**

A total of 117 former baseball players participated (age: 36.8 [13.7] years). The mean dominant SANE score was 70.2 (standard deviation 24.1), and the mean nondominant SANE score was 85.2 (standard deviation 19.4). There was no difference in dominant arm SANE scores when stratified by arm injury history (4.6 [95% confidence interval: −14.9, 5.8]) or arm surgery history (−3.8 [95% confidence interval: 13.4, 5.8]). The shoulders had the greatest persistent joint pain prevalence (28% of all participants) and elbows (21% of all participants). There was no relationship between dominant arm pain or function and playing position.

**Conclusion:**

This is the first study to demonstrate an increase in dominant arm disability in former baseball players. The high prevalence of persistent arm pain and poor arm function among former baseball players is concerning considering participants were younger than 40 years of age. No differences were observed in arm function when stratifying by arm history, surgery, or position demonstrating the potential relationship between baseball participation and arm disability after cessation of play. Clinicians should consider working with baseball players to develop long-term strategies to maintain joint health, especially in the throwing arm, when baseball players are transitioning to retirement. Future research is needed to understand the long-term effectiveness of clinical treatments and the implications of specific arm injuries such as ulnar collateral ligament tears on persistent arm pain and function.

Baseball injuries remain a significant burden,^17^^,^^21^^,^^25^ with a high healthcare cost,^21^^,^^25^ resulting in a large number of workplace days lost,^18^^,^^31^ and this can increase the risk of developing long-term morbidity and/or secondary osteoarthritis.^42^^,^^44^ Post-traumatic osteoarthritis ensues at a younger age, has a greater disability length, and has fewer available treatments compared with idiopathic osteoarthritis.^6^^,^^9^^,^^40^, ^41^ Post-traumatic osteoarthritis has a greater prevalence in former sport participants compared with the general population^9^^,^^43^ and can present as persistent joint pain.^27^ Persistent joint pain can inhibit physical function and activity participation^1^^,^^38^ and is the underlying reason that many individuals seek osteoarthritis medical treatment.^27^ As a result, clinicians and other stakeholder groups have sought to understand the burden of persistent joint pain in different populations.^7^^,^^8^^,^^13^

Baseball is an overhead sport that requires the arm to propel a baseball at high velocities.^14^ Owing to these physical demands, the greatest injury incidence is to the shoulder and elbow.^18^^,^^31^ Different baseball positions have demonstrated disparate arm and body part injury incidence,^31^ potentially demonstrating the different physical requirements as per position. Playing position may be associated with the prevalence of persistent shoulder and elbow pain (a marker of osteoarthritis) in former baseball players, although this has not been investigated. A study in former cricket players found that cricket bowlers had three times greater odds of reporting shoulder persistent pain compared with batters.^8^ Understanding the relationship between persistent joint pain and playing position can allow clinicians to inform stakeholders of the risk and benefits of participating in baseball. Furthermore, such knowledge has potential to assist clinicians in creating personalized physical examinations, interventions, and long-term joint health strategies such as providing increased prevalence of musculoskeletal examinations, providing physical therapy, or counseling to empower individuals to maintain joint health and physical activity levels for baseball players as they transition to retirement.^12^^,^^22^^,^^39^

Currently, the majority of research investigating joint pain related to sport participation has concentrated on soccer, cricket, and rugby.^1^^,^^19^^,^^38^ For example, professional soccer players reported greater lower-extremity persistent pain compared with controls.^1^^,^^38^ However, baseball is one of the most popular sports worldwide with more than112 countries participating.^3^^,^^20^ Furthermore, most research has focused on professional athletes, which have greater training and competition demands and potentially greater sport exposure, compared with lower playing standards.^30^ The paucity of persistent pain research in baseball limits the generalizability of findings and inhibits clinical decisions and education for baseball athletes. Further, the lack of persistent pain research within former baseball players decreases our understanding of former baseball player long-term health, as persistent pain can have harmful effects on physical function and activity participation.^1^^,^^38^ Understanding the relationship between persistent joint pain and baseball participation can inform use of resources to improve musculoskeletal health after retirement from baseball.

The purpose of this study was to (1) evaluate persistent joint pain and arm function in former baseball players and (2) determine whether playing position is associated with increased odds of joint pain and are related to reduced arm function in former baseball players.

## Materials and methods

### Study design

This study used a cross-sectional design that was a subset of a larger study of current and former baseball players. This study included former baseball players who had played at the collegiate and professional levels. This study was approved by the Wake Forest School of Medicine Institutional Review Board.

### Participants and recruitment

**Figure 1:**
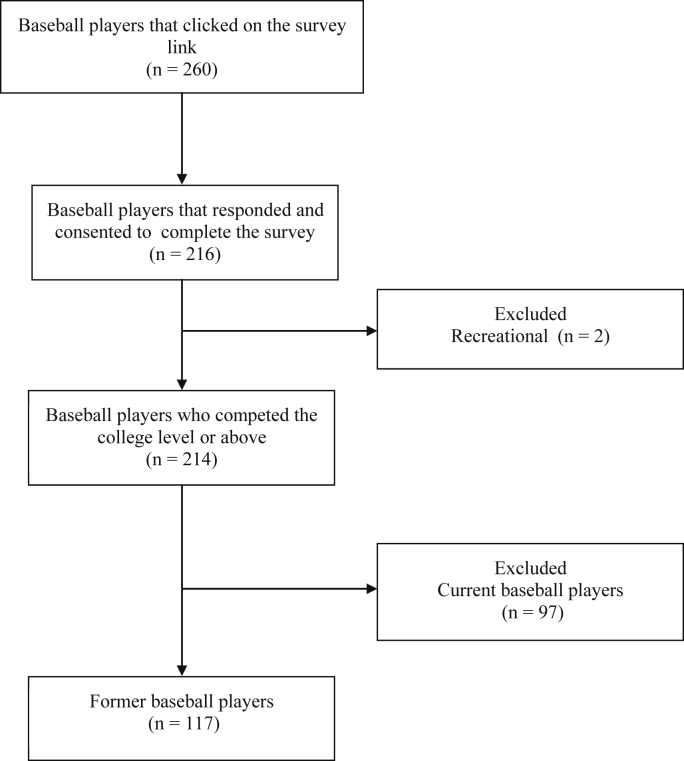
Study participant flow chart.

Participants were recruited from college baseball teams, college baseball team alumni networks, professional baseball organizations, and social media. Recruitment was performed from September 2019 through April 2020. Study consent was obtained on a survey link (Supplementary Appendix S1: Appendix 1) which provided a copy of the participant information sheet and the institutional review board consent form before completing the questionnaire. A total of 260 participants accessed the survey link; of whom, 216 consented to participate (Fig. 1). Eligibility criteria consisted of (1) played ≥1 collegiate baseball season, (2) aged ≥18 years, and (3) formerly played baseball (currently retired from baseball).

### Questionnaire design

The questionnaire was designed to capture five aspects of health and well-being: (i) baseball-related injury; (ii) joint pain and osteoarthritis; (iii) general health and disease prevalence; (iv) physical activity; and (v) resilience, quality of life, and flourishing.^7^^,^^8^ The questionnaire was adopted from a cricket health and well-being study^7^^,^^8^ and was piloted and refined on a group of current and former baseball players (n = 4), collegiate and professional baseball coaches (n = 5), and medical professionals (sport physician, physical therapists, and athletic trainers; n = 4) who specialize in treating baseball players. All responses captured in REDCap software^23^ where participants could save their progress and return to complete at a later time.

### Outcomes

#### Persistent joint pain

Persistent joint pain was assessed with the following question, *“Do you currently experience pain, discomfort, or have any problems in any of your joints?”* If yes, participants were then asked *“Have you had pain in your [left/right] hip/groin, knee, ankle, foot, shoulder, elbow, hand/finger, spine/back, other joint on most days of the last month?*” All participants who recorded persistent pain in the “other joint” category were hand searched for references of wrist or toe persistent joint pain. These data were then recorded as new categories. Wrist persistent pain was categorized with hand/finger, and toe persistent pain was added to foot persistent joint pain.

#### Single Assessment Numeric Evaluation

The Single Assessment Numeric Evaluation (SANE) has been used to evaluate shoulder and elbow overall pain and function. The SANE was evaluated with the following question, “How would you rate your dominant shoulder today as a percentage of normal (0% to 100% scale with 100% being normal)?” and “How would you rate your non-dominant shoulder today as a percentage of normal (0% to 100% scale with 100% being normal)?” ^11^ The SANE is scored on a scale of 0 to 100, with 0 demonstrating full disability and 100 demonstrating no pain and full function.^11^ The SANE has previously been observed to have high validity in patients with shoulder injuries,^45^ patients with osteoarthritis,^15^ arthroplasty,^16^ military cadets,^11^ patients who have undergone shoulder surgery, patients with shoulder anterior instability,^15^ and in athletes after elbow surgery.^2^ The SANE has also demonstrated a high correlation with the Rowe score, the American Shoulder and Elbow Surgeons Rating Scale^11^ and the Constant score.^15^ The minimal clinical important difference in patients with bicep tenodesis was observed to be 10-15 points.^32^

### Explanatory variable

#### Playing position

Playing position was assessed with the following question, *“What is/was your main position of play?”* Response items included pitcher, position player, or two-way player (pitcher and position player). Playing position was determined only from participant report and not from collegiate and professional records.

### Confounders

Confounders were identified through clinical reasoning and literature search.

#### Playing standard

Standard of play was assessed with the following question, “*What was the highest standard of baseball that you played for at least one season*?” Response options included Olympic or World Baseball Classic; Major League Baseball; Minor League Baseball; Independent Baseball; College (4 year), Junior College, High School; Recreation; Don’t know. Participants were stratified into professional (Olympic or World Baseball Classic; Major League Baseball; Minor League Baseball; Independent Baseball) and college (College [4 year], Junior College). Participants who reported *“Don’t know”* were excluded from all analyses.

#### Injury history

Injury history was assessed with the following question, “*Have you ever had any baseball-related injuries leading to more than 4 weeks of reduced participation in exercise, training or sport?”* Response items included *yes, no,* and *don’t know. Don’t know* responses were excluded from analyses. If answered yes, participants were then asked *“Please write the number of injuries for each joint and side for [left/right] hip/groin, knee, ankle, foot, shoulder, elbow, hand/finger, spine/back, other joint.”*

#### Baseball seasons played

Baseball seasons played was assessed with the following question, *“Approximately how many seasons have you played baseball for?”*

Other confounders included age,^26^ body mass index,^29^ handedness,^10^^,^^36^^,^^42^ and orthopedic surgical history.

### Statistical analyses

All data were assessed for missingness before analyses. Missing data were calculated as total and percentage of total data (Supplementary Appendix S2: Appendix 2). Missing data were varied (playing status: 0%; age: 3%; handedness: 0%; body mass index: 3%; joint injury: 7%; SANE: 13%). Missing data were determined to be missing at random. Multiple imputations with chained equations were performed with 100 imputations using the *MICE* package.^46^ Results from the multiple imputations were pooled using Rubin’s rules.^46^ Imputed data were evaluated for consistency and convergence through visual inspection and comparing descriptors and regression coefficients between complete case and imputed data (Supplementary Appendix S2: Appendix 3 and 4). Sensitivity analyses were performed to compare results after complete case analysis and imputed data analysis, which demonstrated similar results (Supplementary Appendix S2: Appendix 5).

Data were reported as mean (standard deviation), median (interquartile range), or count (percentage). SANE scores were then stratified by arm injury and arm surgery history. Analysis of covariance was performed to assess potential differences between dominant arm SANE scores and dominant arm injury history and dominant arm surgery history. Analysis of covariance was adjusted for age, body mass index, arm dominance, playing standard, and years played baseball.

Multivariable logistic and linear regressions were performed to evaluate the relationship between arm persistent pain, arm function, and playing position. Unadjusted and adjusted odds ratios and coefficients and 95% confidence intervals (95% CIs) were calculated. Models were adjusted for age, body mass index, arm dominance, playing standard, years played baseball, and injury and surgery history. All assumptions for logistic and linear regression were evaluated and satisfied.^34^ Sensitivity analyses explored shoulder and elbow persistent joint pain separately, whereby joint injury and surgery history variables were joint specific. Furthermore, owing to the wide CI demonstrated with the arm persistent pain analyses, a Firth corrected logistic regressions was performed to assess the potential for quasi variable separation. All analyses were performed in R, version 4.02 (R Core Team (2013). R: A language and environment for statistical computing. R Foundation for Statistical Computing, Vienna, Austria. URL http://www.R-project.org/), using the *dplyr* package^17^ for cleaning and coding, the *naniar* package for missingness assessment,^28^ and *ggplot2* package^44^ for data visualization.

## Results

A total of 117 former baseball players participated. The mean dominant SANE score was 70.2 (19.4) compared with the mean nondominant SANE score of 84.2 (19.4). The median difference between dominant and nondominant SANE scores was −20.0 (−31.5, −7.5) (Table I).

**Table I:** Descriptive statistics.

Variable	All former baseball players (n = 117)	No persistent joint pain (n = 55)	Current persistent joint pain (n = 62)
Age (yr)	36.8 (13.7)	33.5 (10.6)	38.9 (15.3)
Body mass index (kg/m^2^)	28.0 (3.4)	27.1 (3.2)	28.5 (3.5)
Hand dominance			
Left	23 (20%)	9 (16%)	14 (23%)
Right	94 (80%)	46 (84%)	48 (77%)
Baseball seasons played	17.8 (6.1)	18.2 (7.6)	17.6 (5.6)
Position			
Pitcher	46 (39%)	23 (42%)	23 (37%)
Position player	44 (38%)	21 (38%)	23 (37%)
Two way	27 (23%)	11 (20%)	16 (26%)
Highest standard of play			
College	83 (71%)	37 (67%)	47 (76%)
Professional	33 (29%)	18 (33%)	15 (24%)
History of orthopaedic surgery	60 (51%)	22 (44%)	38 (61%)
History of 4+ wk time loss injury	73 (62%)	34 (62%)	40 (65%)
SANE (dominant arm)	70.2 (24.1)	78.7 (16.6)	63.5 (27.9)
SANE (nondominant arm)	84.2 (19.4)	94.6 (8.30)	76.7 (22.6)

*SANE*, Single Assessment Numeric Evaluation.

Descriptive statistics are reported as mean (standard deviation) or count (percentage).

Persistent pain is defined as having joint pain for “most days of the last month.”

### Comparison of arm function between former baseball players throwing arm injury and surgery history

Former baseball players with a history of arm injury mean SANE score was 68.1 (23.0) and without a history of arm injury mean SANE score was 72.0 (24.8). Former baseball players with a history of dominant arm surgery mean SANE score was 67.5 (22.8) and without a history of dominant arm surgery mean SANE score 71.7 (24.6) (Table II). There was no difference in unadjusted (−4.2 (95% CI: −14.2, 5.8), *P* = .412) or adjusted (−4.6 (95% CI: −14.9, 5.8), *P* = .441) former baseball dominant SANE scores when comparing dominant arm injury history. There was no difference in unadjusted (−3.9 [95% CI: −13.3, 5.6], *P* = .423) or adjusted (−3.8 [95% CI: 13.4, 5.8], *P* = .461) former baseball dominant SANE scores when comparing dominant arm surgery history.

**Table II:** Former baseball player dominant arm function stratified by dominant arm injury and surgery history.

Variable	No dominant arm injury history (n = 68)	Dominant arm injury history (n = 49)	No dominant arm surgical history (n = 82)	Dominant arm surgical history (n = 35)
SANE (dominant arm)	72.0 (24.8)	68.1 (23.0)	71.7 (24.6)	67.5 (22.8)
Age (yr)	37.7 (15.8)	35.3 (10.5)	36.9 (14.6)	36.0 (11.9)
Body mass index (kg/m^2^)	27.9 (3.7)	28.0 (3.0)	28.2 (3.6)	27.4 (2.9)
Hand dominance				
Left	10 (15%)	13 (27%)	14 (17%)	9 (26%)
Right	58 (85%)	36 (73%)	68 (83%)	26 (74%)
Baseball seasons played	17.1 (5.7)	18.5 (6.5)	16.5 (4.9)	20.4 (7.6)
Position				
Pitcher	23 (35%)	23 (47%)	28 (34%)	18 (51%)
Position player	30 (44%)	14 (29%)	36 (44%)	8 (23%)
Two way	15 (22%)	12 (24%)	18 (22%)	9 (26%)
Highest standard of play				
College	53 (78%)	31 (63%)	61 (74%)	23 (66%)
Professional	15 (12%)	18 (37%)	21 (26%)	12 (34%)

*SANE*, Single Assessment Numeric Evaluation.

Descriptive statistics are reported as mean (standard deviation) or count (percentage).

### Persistent pain prevalence

Fifty-three percent of all former baseball players reported having persistent joint pain in at least one joint, and 30% reported persistent joint pain in two or more joints. The greatest persistent joint pain prevalence was at the shoulders (28% of participants), with 94% of persistent joint pain occurring in the throwing shoulder. Twenty-one percent of participants reported persistent elbow pain, all (100%) of which were attributed to the throwing elbow (Table III). When comparing by position, pitchers reported the greatest persistent pain prevalence to the throwing shoulder (33%), with 15% reported elbow persistent pain. Position players reported the greatest persistent pain prevalence to the throwing elbow (25%) and throwing shoulder (23%) and two-way players reported the greatest persistent pain prevalence to the throwing shoulder (26%) and throwing elbow (26%) (Table IV).

**Table III:** Former baseball players (n = 117) persistent joint pain.

	Count (%)
Persistent joint pain (1+ joints)	62 (53)
Persistent joint pain (2+ joints)	35 (30)
Shoulder	34 (29)
Throwing shoulder	32 (28)
Elbow	25 (21)
Throwing elbow	25 (21)
Hand/Finger persistent pain	9 (8)
Neck persistent pain	7 (6)
Midback persistent pain	5 (4)
Low back persistent pain	14 (12)
Hip persistent pain	9 (8)
Knee persistent pain	30 (26)
Ankle persistent pain	6 (5)
Foot/Toe persistent pain	12 (10)

Persistent pain is defined as having joint pain for “most days of the last month.”

**Table IV:** Comparison of pitchers, position players, and two-way players for persistently painful joints with the highest prevalence.

Persistent painful joint	Pitcher (n = 46)	Position player (n = 44)	Two-way player (n = 27)
Throwing shoulder	15 (33%)	10 (23%)	7 (26%)
Throwing elbow	7 (15%)	11 (25%)	7 (26%)
Knee	15 (33%)	10 (23%)	5 (19%)
Low back	5 (11%)	5 (11%)	4 (15%)

### The relationship of playing position on persistent arm pain and function

Playing position (two-way: 12.30 [0.50, 30.0], *P* = .130; Pitcher: 9.20 [0.70, 11.9], *P* = .095; Table V) had similar odds of reporting persistent arm pain in former baseball players. Sensitivity analyses demonstrated similar results. The Firth corrected logistic regression demonstrated no adjusted association between persistent arm pain and playing position (Supplementary Appendix S2: Appendix 6). There was no association between elbow or shoulder persistent pain playing position in the adjusted analysis (Supplementary Appendix S2: Appendix 7). Another sensitivity analysis observed that pitchers reported an increased odds of arm persistent joint pain (4.36 [1.28, 11.9]) compared to non-pitchers (two-way and position players combined; Supplementary Appendix S2: Appendix 8).

**Table V:** The odds and relationship of playing position and arm pain and arm function in former baseball players.

Variable	Persistent shoulder/elbow pain	SANE
	Odd ratios (95% CI)	Beta (95% CI)
Crude (unadjusted)		
Two way∗	0.28 (0.05, 1.61) *P* = .157	−0.15 (−12.11, 11.81), *P* = .839
Pitcher∗	1.82 (0.40, 8.24) *P* = .439	−0.08 (−14.43, 14.59), *P* = .974
Adjusted		
Two way∗	12.30 (0.50, 30.0), *P* = .130	−3.61 (−18.21, 11.00), *P* = .802
Pitcher∗	9.20 (0.70, 11.9), *P* = .095	0.11 (−16.24, 16.45), *P* = .843
Professional†	1.38 (0.71, 2.67), *P* = .342	1.03 (−3.03, 5.10), *P* = .996
Baseball seasons	1.12 (0.99, 1.23), *P* = .062	−0.08 (−0.99, 0.84), *P* = .717
History of throwing arm joint injuryΦ	1.04 (0.15, 8.51), *P* = .917	−3.66 (−16.67, 9.33), *P* = .402
Age	1.04 (0.97, 1.11), *P* = .306	−0.31 (−0.72, 0.11), *P* = .244
Body mass index	1.05 (0.79, 1.41), *P* = .720	−0.60 (−2.45, 1.23), *P* = .162
Right handed‡	0.01 (0.001, 0.09), *P* = .001	6.89 (−8.29, 22.07), *P* = .869
History of throwing arm orthopedic surgeryΘ	1.70 (0.27, 10.6), *P* = .572	−0.07 (−13.91, 13.78), *P* = .658

*SANE*, Single Assessment Numeric Evaluation; *95% CI*, 95% confidence interval.

Persistent pain was defined as pain on “*most days of the last most.*”

∗ Position players were used as the reference category in the multivariable analyses.

† College baseball players were used as the reference category in the multivariable analyses.

‡ Left handed players were used as the reference category in the multivariable analyses.

There was no relationship between dominant arm SANE score and playing position (two-way: −3.61 [−18.21, 11.00], *P* = .802; Pitcher: 0.11 [−16.24, 16.45], *P* = .843) (Table V). Sensitivity analyses demonstrated similar results when comparing pitchers to nonpitchers (Supplementary Appendix S2: Appendix 8).

## Discussion

The most important result of our study was despite the relatively young age of the cohort (37 years), their mean dominant arm SANE score was 70 compared with their nondominant arm SANE score of 85. Furthermore, players who reported persistent joint pain in any joint, their SANE score was 63. There were no differences in dominant arm SANE scores between former baseball players with or without a history of throwing arm injury or throwing arm surgery. These dominant arm SANE scores are lower than the average or median score reported by older anatomic total shoulder arthroplasty (median 90), reverse total shoulder arthroplasty (median 85), rotator cuff repair (mean 75), or other shoulder patients (mean 80) after treatment with SANE scores.^4^ Not surprisingly, shoulder pain was the most prevalent, with elbow persistent pain reported as the third most prevalent. Ninety-six percent of all shoulder and elbow persistent pain was to the throwing arm. After controlling for confounders, playing position was not related to persistent arm pain or arm function in former baseball players.

Former baseball players reported a mean dominant SANE arm function score of 70 compared with a mean nondominant SANE arm score of 85. These dominant arm scores are similar to a US general population sample (mean age of 52.6 years), after treatment of pathologic shoulder conditions (mean score: 70.8). Furthermore, these dominant arm SANE scores were worse (exceeded the minimal clinical important difference of 10-15 points)^32^ than a sample of patients with total shoulder arthroplasty (mean age 68.9 years) at two year after surgery (mean score: 90).^4^ Dominant arm function reported by this sample of former baseball players is concerning. This sample is younger and is not a shoulder surgical or injury cohort in comparison with past literature.^4^^,^^33^ Furthermore, these dominant SANE scores are on average 15 points lower compared with their nondominant SANE scores, which is greater than the minimal clinical important difference, demonstrating a clinically significant difference.^32^ When comparing by injury and surgery history, before and after controlling for confounders, there was no difference in SANE scores between former baseball players with or without a history of arm injury or arm surgery. The diminished dominant arm SANE scores reported by this sample are potentially affected by the high persistent shoulder and elbow pain prevalence.^11^ These findings suggest high-level baseball exposure at the collegiate or professional level, beyond injury or surgical history, may contribute to diminished throwing arm function after retirement from baseball. Thus, former baseball players may require specific throwing arm examinations and potential interventions, such as physical therapy and surgery,^4^^,^^33^ to improve overall arm function. Sports medicine clinicians should educate baseball players of the risk of decreased arm function after retirement.

The highest persistent joint pain prevalence was at the shoulder and elbow and this was similar when compared by position. This is in contrast to research in former cricket and soccer players in which persistent joint pain was most prevalent in the knee, back, and hand.^8^^,^^38^ These differences may be owing to the specific sport tasks and physical requirements. Both cricket and baseball are overhead throwing sports,^14^^,^^37^ while soccer is a field sport, which primarily involves the lower extremity.^5^ Differences in baseball and cricket shoulder and elbow persistent joint pain prevalence may be owing to the dissimilar throwing biomechanics used by both sports.^14^^,^^35^ Baseball uses a bent elbow during the late cocking throwing phase, increasing shoulder and elbow forces, and subsequently shoulder and elbow injuries.^14^ In contrast, cricket bowling requires a straight arm, using increased spinal extension and rotation to propel the cricket ball, increasing spinal forces, and subsequent spinal injuries.^35^ More than 95% of all shoulder and persistent elbow pain was to the throwing arm. The increased forces to the shoulder and elbow during the baseball throwing motion may increase persistent throwing arm pain; however, further prospective longitudinal studies are required to understand this potential relationship.

There was also no relationship between playing position and persistent arm pain or arm function and pitchers, position players and two-way players reported similar persistent joint prevalence to the throwing shoulder. It should be noted that the wide CIs reported for playing position may demonstrate a potential relationship with persistent joint pain; however, the sample size may have hindered the precision in these analyses. To potentially control for the decreased sample size, a Firth corrected sensitivity analysis was performed, which also reported similar odds ratios. Furthermore, owing to two-way players during the athlete public involvement sessions reporting these players primarily played a position and only pitched on minor occasions, another sensitivity analysis was performed. The sensitivity analyses investigating the relationship between pitchers and nonpitchers demonstrated increased odds of persistent joint pain in pitchers compared with nonpitchers but similar results for reported arm function. These results should also be interpreted with caution owing to the wide CIs. While different baseball positions require different skills and physical attributes, all positions require high levels of throwing.^24^ There is also the potential for residual confounding. As baseball players usually play multiple positions at younger ages, grouping player by college and professional position may only partially control for this explanatory variable. For example, baseball players that played outfield and pitcher through high school, but only outfield at the college and professional level would only be grouped as a position player within this study. As high school pitchers can pitch over 100 innings per year, this grouping would miss significant pitching exposure. Despite these issues, the overall similarities in baseball participation between positions may incur similar risks for persistent joint pain and arm function. Baseball playing positions should potentially be given equal weight when examining former baseball player’s joint health and arm function.

These findings suggest future research, with this study serving as a foundation to begin to understand former baseball participation and long-term health. This study observed that the former baseball player dominant arm mean SANE score was 70 compared with the nondominant arm mean SANE score of 85, despite the average age of this cohort of 37 years, and 96% of all shoulder and elbow persistent pain was to the throwing arm. These findings suggest that former baseball players exhibit particular musculoskeletal persistent pain and function phenotypes compared with other former professional sports^8^^,^^38^ and the US general population.^4^^,^^33^ Implementing larger samples with appropriate controls can help clinicians determine the long-term effectiveness of clinical treatments on arm health and clinical recommendations for baseball players as they transition into retirement. Furthermore, as ulnar collateral ligament injuries continue to rise,^17^^,^^21^^,^^25^ discerning the unique long-term health implications of this injury can help improve clinical short- and long-term interventions.

### Strengths and potential limitations

This study used previously validated questions and clinical outcome measures to assess persistent joint pain, arm health, and function,^4^^,^^7^^,^^8^ allowing for an improved ability to compare these results to past literature. Key stakeholder involvement was incorporated into the survey development process which increased the applicability of this study. The recruitment methodology used does not allow for a true response rate to be calculated, resulting in an inability to understand selection bias. Using a cross-sectional design requires participants to recall past events, potentially resulting in recall bias, decreasing the precision of these results. Participants reported playing position by what position they identified as. Some players may have played a primary position, but switched to another position (ie, catcher to pitcher) at a higher playing standard, which decreases the precision of these results. There are differences in etiology between elbow and shoulder injuries and surgery. While arm injury and orthopedic surgery history was controlled for within the analyses, there is still residual confounding that can bias these results toward the null. Using one data collection method potentially increases the risk of single method bias, which may deter data collection within individuals with poor computer literacy skills or access to technology. Persistent joint pain prevalence was assessed; however, pain severity was not calculated. As pain severity may be a marker of specific osteoarthritis lesions,^37^ this decreases the clinical utility of these findings. The diminished dominant arm SANE score may be influenced by participant internal control. Former baseball players in this sample participated at the collegiate and professional ranks, demonstrating high skill and proficiency in throwing. These former baseball players may report poor dominant arm SANE scores owing to the inability to throw or play baseball at a high level, compared with current daily arm function.

## Conclusions

The high prevalence of persistent dominant arm pain and poor dominant arm function among former baseball players remains a public health concern. There was also no observed relationship between persistent dominant arm pain or function and playing position. These findings potentially suggest that high level baseball participation is related to increased dominant arm pain and reduced dominant arm function following cessation of play. Clinicians should consider educating patients and implementing strategies to maintain joint health, especially in the throwing arm, when baseball players are transitioning to retirement. These findings highlight the need for further research into the unique long-term health implications of baseball participation, with specific inquiries into arm health, the effectiveness of clinical treatments, and the implications of specific arm injuries such as ulnar collateral ligament tears.

## Disclaimers

*Funding:* Centre for Sport, Exercise and Osteoarthritis Research Versus Arthritis Grant (grant reference 21595). Gary Collins was supported by the NIHR Biomedical Research Centre, Oxford.

*Conflicts of interest:* The authors, their immediate families, and any research foundations with which they are affiliated have not received any financial payments or other benefits from any commercial entity related to the subject of this article.
