# Supplementary material for: Persistent joint pain and arm function in former baseball players
Source: JSES Int. 2021 Jun 29;5(5):912–9. doi: 10.1016/j.jseint.2021.05.001 (PMC8411053; doi:10.1016/j.jseint.2021.05.001)
Supplement: Supplementary Appendix S1 [file mmc1.docx]

**Appendix 1.** Questionnaire

# **1. Personal details and general health**

| 1.1. **Gender:** | Male |  | Female |  | Other |  |
| --- | --- | --- | --- | --- | --- | --- |

| 1.2. **Age:** | _______  M M Y Y Y Y |
| --- | --- |

| 1.3. **Weight:** |  |  | kg |  | pounds |  |  |
| --- | --- | --- | --- | --- | --- | --- | --- |

| 1.4. **Height:** |  |  | cm |  | Feet/inches |  |  |
| --- | --- | --- | --- | --- | --- | --- | --- |

# **2. Baseball history**

| 2.1. **Playing status:** | Currently playing |  | No longer |  | Plan to return |  |
| --- | --- | --- | --- | --- | --- | --- |
|  | baseball |  | playing baseball |  | to baseball |  |

| 2.2. **What age were you when you played your *first* baseball game?**   \|  \| (years old) \| Don’t know \|  \| \| --- \| --- \| --- \| --- \| |  |
| --- | --- | --- | --- | --- | --- |
|  |  |

2.3. **What was your main position(s) of play?** (select all that apply)**:**

| Catcher |  |  |  | Position Player | | |  |  |  |  |
| --- | --- | --- | --- | --- | --- | --- | --- | --- | --- | --- |
|  |  |  |  | Please Specify Position | | | | _______________________ | | |
| Pitcher |  |  |  | Other | | |  |  |  |  |
| Starter |  | Reliever |  | Please Specify | __ |  |  |  |  |  |

2.4. **Do you throw with your left or right hand?**

| Left |  | Right |  | Both |  |
| --- | --- | --- | --- | --- | --- |

2.5. **Approximately how many seasons have you played baseball for?**

|  | Number of seasons: |  |
| --- | --- | --- |

2.6. **What was the highest level of baseball that you played for at least one season? (please select only one)**

| Major League Baseball |  |  | Independent League |  |
| --- | --- | --- | --- | --- |
|  |  |  |  |  |
| Represented country |  |  | College |  |
| at an international |  |  |  |  |
| competition |  |  | High School |  |
| (i.e. Olympics, World |  |  |  |  |
| Baseball Classic, etc.) |  |  | Recreational (i.e. Adult |  |
|  |  |  | League, Babe Ruth, Little | |
| Minor League |  |  | League, etc.) |  |
|  |  |  |  |  |
| International Professional |  |  | Don’t know |  |
| League ( i.e. Japan |  |  |  |  |
| Korea, Mexico, etc.) |  |  |  |  |

# **3. Pain, injury and surgery**

3.1. **Have you ever had orthopedic surgery (including bone, ligament or joint surgery)?**

| Yes |  | No |  |
| --- | --- | --- | --- |

3.2. **If yes, where? Please write the number of surgeries for each joint and side**

(e.g. Hip left (L / 3, right (R / 0)

| **Hip** | |  | |  |  |  |  | | | **Shoulder** | |  |  |  |  |
| --- | --- | --- | --- | --- | --- | --- | --- | --- | --- | --- | --- | --- | --- | --- | --- |
| Number of surgeries: | | **L** | |  | **R** |  |  | | | Number of surgeries: | | **L** |  | **R** |  |
|  | |  | |  |  |  |  | | |  | |  |  |  |  |
| **Knee** | |  | |  |  |  |  | | | **Elbow** | |  |  |  |  |
| Number of surgeries: | | **L** | |  | **R** |  |  | | | Number of surgeries: | | **L** |  | **R** |  |
|  | |  | |  |  |  |  | | |  | |  |  |  |  |
| **Ankle** | |  | |  |  |  |  | | | **Hand/finger** | |  |  |  |  |
| Number of surgeries: | | **L** | |  | **R** |  |  | | | Number of surgeries: | | **L** |  | **R** |  |
|  | |  | |  |  |  | |  | |  | |  |  |  |  |
| **Spine/back** | |  | |  |  |  | |  | | **Face/Jaw** | |  |  |  |  |
| Number of surgeries: | |  | |  |  |  | |  | | Number of surgeries: | | **L** |  | **R** |  |
| Upper Back |  | | Middle | | |  | Low Back | |  | |  |  |  |  |  |
|  |  | | Back | | |  |  | |  | |  |  |  |  |  |

Other joint(s), please specify: _____________________________________________

3.3 **Have you ever had a hip replacement?**

| Yes* |  | No |  | Don’t know |  |
| --- | --- | --- | --- | --- | --- |

| 3.4 ***If yes,** **which side was your hip replacement on?** | | | | | | | | |
| --- | --- | --- | --- | --- | --- | --- | --- | --- |
|  |  | Left |  | Right |  | Both sides |  |  |

3.5 **Have you ever had a knee replacement?**

| Yes* |  | No |  | Don’t know |  |
| --- | --- | --- | --- | --- | --- |

| 3.6 ***If yes,** **which side was your knee replacement on?** | | | | | | | |
| --- | --- | --- | --- | --- | --- | --- | --- |
|  | Left |  | Right |  | Both |  |  |

3.7 **Have you ever had a shoulder replacement?**

| Yes* |  | No |  | Don’t know |  |
| --- | --- | --- | --- | --- | --- |

| 3.8 ***If yes,** **which side was your shoulder replacement on?** | | | | | | | |
| --- | --- | --- | --- | --- | --- | --- | --- |
|  | Left |  | Right |  | Both |  |  |

3.9 **Have you ever had an elbow replacement?**

| Yes* |  | No |  | Don’t know |  |
| --- | --- | --- | --- | --- | --- |

| 3.10 ***If yes,** **which side was your elbow replacement on?** | | | | | | | |
| --- | --- | --- | --- | --- | --- | --- | --- |
|  | Left |  | Right |  | Both |  |  |

3.11 **Have you ever had an Anterior Cruciate Ligament (ACL) Reconstruction:**

| Yes* |  | No |  | Don’t know |  |
| --- | --- | --- | --- | --- | --- |

| 3.12 ***If yes,** **which ACL was reconstructed?** | | | | | | | |
| --- | --- | --- | --- | --- | --- | --- | --- |
|  | Left knee |  | Right knee |  | Both knees |  |  |

3.13 **Have you ever had knee cartilage or meniscus surgery?**

| Yes* |  | No |  | Don’t know |  |
| --- | --- | --- | --- | --- | --- |

| 3.14 ***If yes,** **which knee had cartilage or meniscus surgery?** | | | | | | | |
| --- | --- | --- | --- | --- | --- | --- | --- |
|  | Left knee |  | Right knee |  | Both knees |  |  |

3.15 **Have you ever had Ulnar Collateral Reconstruction (i.e. “Tommy John”) surgery?**

| Yes* |  | No |  | Don’t know |  |
| --- | --- | --- | --- | --- | --- |

| 3.16 ***If yes,** **which elbow had Ulnar Collateral Reconstruction (i.e. “Tommy John”) surgery?** | | | | | | | |
| --- | --- | --- | --- | --- | --- | --- | --- |
|  | Left elbow |  | Right elbow |  | Both elbows |  |  |

3.17 **Have you ever had rotator cuff surgery?**

| Yes* |  | No |  | Don’t know |  |
| --- | --- | --- | --- | --- | --- |

| 3.18 ***If yes,** **which shoulder had rotator cuff surgery?** | | | | | | |
| --- | --- | --- | --- | --- | --- | --- |
|  | Left shoulder |  | Right shoulder |  | Both shoulders |  |

3.18 **Have you ever had labrum or SLAP shoulder surgery?**

| Yes* |  | No |  | Don’t know |  |
| --- | --- | --- | --- | --- | --- |

| 3.19 ***If yes,** **which shoulder had rotator cuff surgery?** | | | | | | |
| --- | --- | --- | --- | --- | --- | --- |
|  | Left shoulder |  | Right shoulder |  | Both shoulders |  |

3.20 **Have you ever had a finger joint replacement?**

| Yes* |  | No |  | Don’t know |  |
| --- | --- | --- | --- | --- | --- |

3.21***If yes,** **which hand did you have the finger joint replacement?**

|  | Left hand |  | Right hand |  | Both hands |  |
| --- | --- | --- | --- | --- | --- | --- |

3.22. **Have you ever had any baseball-related injuries leading to more than *4 weeks* of reduced participation in exercise, training or sport?**

| Yes |  | No |  | Don’t know |  |
| --- | --- | --- | --- | --- | --- |

3.23. **If yes, where? Please write the number of injuries for each joint and side**

**(eg Hip left (L / 3, right (R / 1)**

| **Hip** |  |  |  |  |  | | **Shoulder** |  |  |  |  |  |
| --- | --- | --- | --- | --- | --- | --- | --- | --- | --- | --- | --- | --- |
| Number of Injuries: | **L** |  | **R** |  |  | | Number of Injuries: | **L** |  | **R** |  |  |
|  |  |  |  |  |  | |  |  |  |  |  |  |
| **Knee** |  |  |  |  |  | | **Elbow** |  |  |  |  |  |
| Number of Injuries: | **L** |  | **R** |  |  | | Number of Injuries: | **L** |  | **R** |  |  |
|  |  |  |  |  |  | |  |  |  |  |  |  |
| **Ankle** |  |  |  |  |  | | **Hand/finger** |  |  |  |  |  |
| Number of Injuries: | **L** |  | **R** |  |  | | Number of Injuries: | **L** |  | **R** |  |  |
|  |  |  |  |  | |  |  |  |  |  |  |  |
| **Spine/back** |  |  |  |  | |  | **Face/Jaw** |  |  |  |  |  |
| Number of Injuries: |  |  |  |  | |  | Number of Injuries: | **L** |  | **R** |  |  |

Other joint(s), please specify: _____________________________________________

3.24. **Do you currently experience pain, discomfort, or have any problems in any of your joints?**

| Yes |  | No |  |
| --- | --- | --- | --- |

3.25. **If yes, where? Please select all that apply and indicate which side(s)**

| **Hip/Groin** |  |  |  |  |  | |  |  | **Shoulder** |  |  |  |  |  | | |  | |
| --- | --- | --- | --- | --- | --- | --- | --- | --- | --- | --- | --- | --- | --- | --- | --- | --- | --- | --- |
| Side(s): | **L** |  | **R** |  | **Both** | |  |  | Side(s): | **L** |  | **R** |  | **Both** | | |  | |
|  |  |  |  |  |  | |  |  |  |  |  |  |  |  | | |  | |
| If yes, have you had pain in your Hip/Groin on most days of the last month? | | | | | | **Yes** |  |  | If yes, have you had pain in your Shoulder on most days of the last month? | | | | | | **Yes** |  | |  |
|  |  |  |  |  |  |  |  |  |  |  |  |  |  |  |  |  | |  |
|  |  |  |  |  |  | **No** |  |  |  |  |  |  |  |  | **No** |  | |  |

| **Knee** |  |  |  |  |  | |  |  | **Elbow** |  |  |  |  |  | | |  | |
| --- | --- | --- | --- | --- | --- | --- | --- | --- | --- | --- | --- | --- | --- | --- | --- | --- | --- | --- |
| Side(s): | **L** |  | **R** |  | **Both** | |  |  | Side(s): | **L** |  | **R** |  | **Both** | | |  | |
|  |  |  |  |  |  | |  |  |  |  |  |  |  |  | | |  | |
| If yes, have you had pain in your Knee on most days of the last month? | | | | | | **Yes** |  |  | If yes, have you had pain in your Elbow on most days of the last month? | | | | | | **Yes** |  | |  |
|  |  |  |  |  |  |  |  |  |  |  |  |  |  |  |  |  | |  |
|  |  |  |  |  |  | **No** |  |  |  |  |  |  |  |  | **No** |  | |  |

| **Ankle** |  |  |  |  |  | |  |  | **Hand** |  |  |  |
| --- | --- | --- | --- | --- | --- | --- | --- | --- | --- | --- | --- | --- |
| Sides(s): | **L** |  | **R** |  | **Both** | |  |  | Sides(s): **L**   **R** | **Both** |  |  |
| If yes, have you had pain in your Ankle on most days of the last month? | | | | | | **Yes** |  |  | If yes, have you had pain in your Hand on most days of last month? | **Yes**  **No** |  |  |
|  |  |  |  |  |  |  |  |  |  |  |  |  |
|  |  |  |  |  |  | **No** |  |  |  |  |  |  |

| **Spine** |  |  |  |  |  |  |  | **Face/Jaw** |  |  |  |  |  | | |  | |
| --- | --- | --- | --- | --- | --- | --- | --- | --- | --- | --- | --- | --- | --- | --- | --- | --- | --- |
| Side(s) | **Neck** |  | **Mid** |  | **Low** |  |  | Side(s): | **L** |  | **R** |  | **Both** | | |  | |
|  |  |  |  |  |  |  |  |  |  |  |  |  |  | | |  | |
| If yes, have you had pain in your Spine on most days of the last month? | | | | | **Yes** |  |  | If yes, have you had pain in your Face/Jaw on most days of the last month? | | | | | | **Yes** |  | |  |
|  |  |  |  |  |  |  |  |  |  |  |  |  |  |  |  | |  |
|  |  |  |  |  | **No** |  |  |  |  |  |  |  |  | **No** |  | |  |

| Other joint(s), please specify: |  |
| --- | --- |

3.26. How would you rate your shoulder today as a percentage of normal (0% to 100% scale with 100% being normal)? 0 ------------------------------100 (slide rule within electronic link)
