# Supplementary material for: Persistent joint pain and arm function in former baseball players
Source: JSES Int. 2021 Jun 29;5(5):912–9. doi: 10.1016/j.jseint.2021.05.001 (PMC8411053; doi:10.1016/j.jseint.2021.05.001)
Supplement: Supplementary Appendix S2 [file mmc2.docx]

Supplemental File 2:

Appendix 2. Missing Data

Missing Counts and Percentage

Playing Status: 0, 0%

Age: 12, 6%

Gender: 2, 6%%

Weight: 12, 5.6%

Height: 12, 5.6%

First Game Age: 4, 1.9%

Smoker: 12, 6%

Smokeless Tobacco: 12, 6%

Electronic Cigarettes: 12, 6%

Asthma: 12, 6%

Employment Status: 12, 6%

Education: 12, 6%

SANE: 26, 12%

QOL Impact: 35, 16%

Resilience Question: 35, 16%

Flourish: 38, 17%%

HRQOL: 35, 16%%

IPAQ: 21, 10%

Osteoarthritis: 16, 7%

Current Pain: 16, 7%

Persistent Joint Pain: 16, 7%

Surgery History: 16, 7%

Throwing Hand: 0, 0%

Figure 1. Grouped Missing Data


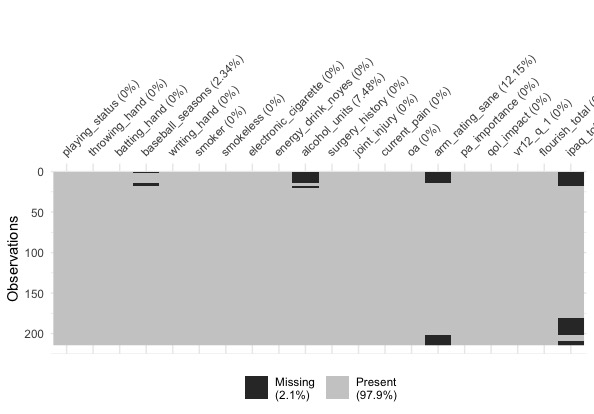


Figure 2. Ungrouped Missing Data


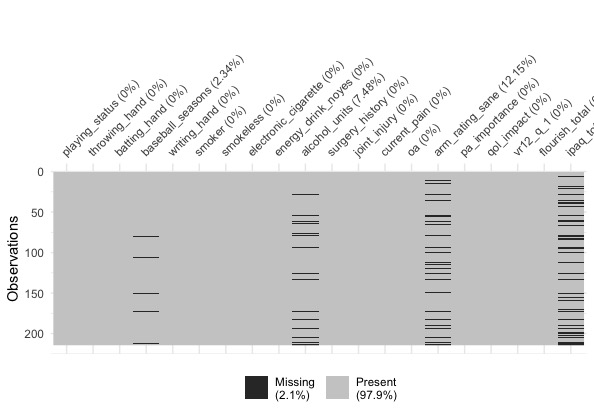


Figure 3. Comparing Missing Data by Current and Former Baseball Players


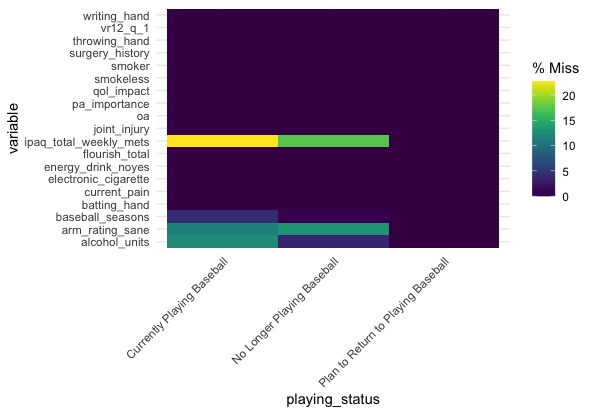


Appendix 3. Imputed Former Baseball Player Descriptive Statistics

| Variable | Former Baseball Players  (n = 117) |
| --- | --- |
| Age (years) | 36.6 (13.7) |
| Body Mass Index (kg/m^2^) | 27.9 (3.4) |
| Hand Dominance  Left  Right | 23 (20%)  94 (80%) |
| Baseball Seasons Played | 17.7 (6.0) |
| Position  Pitcher  Position Player  Two Way | 46 (39%)  44 (38%)  27 (23%) |
| Highest Standard of Play  College  Professional | 83 (71%)  33 (39%) |
| Education  High School Diploma  University Degree | 12 (10%)  0 (0%) |
| High Blood Pressure | 16 (14%) |
| Alcohol Units per Week | 4 (1, 8) |
| Physical Activity Per Week (METS) | 3972 (1698, 6246) |
| Flourishing Scale Score^a^ | 51.2 (6.0) |
| PCS^b^ | 48.9 (6.8) |
| MCS^b^ | 51.8 (8.4) |
| SANE^c^ (Dominant Arm) | 70.3 (24.0) |
| SANE^c^ (Non-Dominant Arm) |  |
| History of Orthopaedic Surgery | 60 (51%) |
| History of ≥4 week time loss injury | 73 (62%) |
| Current Pain | 62 (53%) |

Descriptive statistics are reported as mean (standard deviation), median (interquartile range), or count (percentage)

Data were imputed for 20 iterations.

1 alcohol unit is equivalent to one shot, 4 ounces of wine, or a half pint of beer

METS = Metabolic Equivalents

PCS = Physical Component Score of the VR-12

MCS = Mental Component Score of the VR-12

SANE = Single Assessment Numeric Evaluation

^a^ Flourishing total score is scored from 8 (strong disagreement on all items) to 56 (strong agreement on all items). A higher score is considered greater flourishing.

^b^ Physical Component Scores (PCS) and Mental Component Scores (MCS) were calculated using norm based scoring (population norm 50 SD 10, high scorer = better health-related quality of life)

^c^ The SANE is scored on a scale of 0 to 100, with 0 demonstrating full disability and 100 demonstrating no pain and full function

Appendix 4. Multiple Imputation Convergence Assessment

Figure 1. Single Assessment Numeric Evaluation Imputation Convergence

**
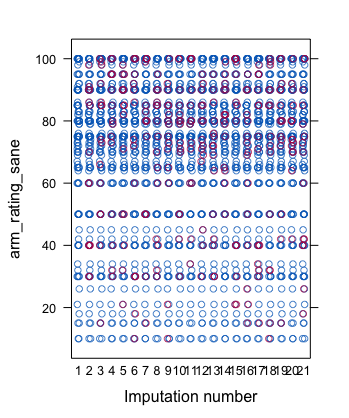
**

Figure 2. Single Assessment Numeric Evaluation Imputation Density

**
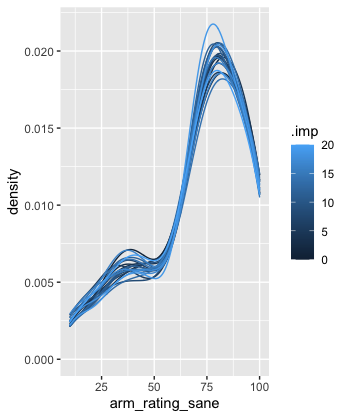
**

Appendix 5. Sensitivity Analysis: The adjusted odds and relationship of playing position association with joint pain, arm pain, and arm function in former baseball players using a complete case analysis

| Variable | Persistent Shoulder/Elbow Pain | SANE |
| --- | --- | --- |
|  | Odds Ratio  (95% CI) | Beta  (95% CI) |
| Two Wayϑ | 25.33 (0.68, 940.87),  p = 0.080 | -4.45 (-19.52, 10.62),  p = 0.704 |
| Pitcherϑ | 16.62 (0.95, 290.32),  p = 0.05 | 2.46 (-18.67, 15.47),  p = 0.704 |
| Professional* | 4.56 (0.50, 41.62),  p = 0.178 | -0.11 (-13.18, 13.41),  p = 0.860 |
| Baseball Seasons | 1.16 (1.01, 1.34),  p = 0.039 | -0.07 (-1.00, 0.85),  p = 0.947 |
| History of Joint InjuryΦ | 3.21 (0.37, 28.07),  p = 0.292 | -3.75 (-17.27, 9.76),  p = 0.706 |
| Age | 1.05 (0.97, 1.13),  p = 0.212 | -0.36 (-0.82, 0.09),  p = 0.105 |
| Body Mass Index | 0.99 (0.72, 1.37),  p = 0.979 | -0.39 (-2.29, 1.52),  p = 0.302 |
| Right Handedψ | - 1. (0.001, 0.09),   p = 0.001 | 5.03 (-10.82, 20.87),  p = 0.608 |
| History of Orthopaedic SurgeryΘ | 0.39 (0.04, 3.84),  p = 0.421 | -1.52 (-15.94, 12.89),  p = 0.576 |

SANE = Single Assessment Numeric Evaluation

95% CI = 95% Confidence Interval

Persistent pain was defined as pain on ‘*most days of the last most’*

ϑ Position players were used as the reference category in the multivariable analyses

* College baseball players were used as the reference category in the multivariable analyses

ψ Left handed players were used as the reference category in the multivariable analyses

Φ For the persistent joint injury analyses, this is referring to a history of any joint injury, in contrast to the arm persistent pain and SANE analyses, this is referring to a history of a throwing arm joint injury

ΘFor the persistent joint injury analyses, this is referring to a history of any orthopaedic surgery, in contrast to the arm persistent pain and SANE analyses, this is referring to a history of a throwing arm orthopaedic surgery

Appendix 6. Sensitivity Analysis: The Firth corrected adjusted odds of playing position and persistent arm pain in former baseball players

| Variable | Persistent Joint Pain |
| --- | --- |
|  | Odds Ratio  (95% CI) |
| Two Wayϑ | 6.72 (0.54, 83.1),  p = 0.142 |
| Pitcherϑ | 6.44 (0.73, 57.2),  p = 0.098 |
| Professional* | 1.24 (0.72, 2.15),  p = 0.430 |
| Baseball Seasons | 1.12 (1.01, 1.24),  p = 0.043 |
| History of Joint InjuryΦ | 2.24 (0.40, 12.6),  p = 0.361 |
| Age | 1.04 (0.98, 1.09),  p = 0.196 |
| Body Mass Index | 0.98 (0.76, 1.26),  p = 0.849 |
| Right Handedψ | 0.01 (0.001, 0.16),  p < 0.001 |
| History of Orthopaedic SurgeryΘ | 0.50 (0.08, 3.35),  p = 0.478 |

95% CI = 95% Confidence Interval

Persistent pain was defined as pain on ‘*most days of the last most’*

ϑ Position players were used as the reference category in the multivariable analyses

* College baseball players were used as the reference category in the multivariable analyses

ψ Left handed players were used as the reference category in the multivariable analyses

Φ History of a throwing arm joint injury

Θ History of a throwing arm orthopaedic surgery

Appendix 7. Sensitivity Analysis: The adjusted odds of playing position and elbow or shoulder joint pain in former baseball players

| Variable | Persistent Elbow Joint Pain | Persistent Shoulder Joint Pain |
| --- | --- | --- |
|  | Odd Ratios  (95% CI) | Odd Ratios  (95% CI) |
| Adjusted | | |
| Two Wayϑ | 1.78 (0.48, 6.57),  p = 0.386 | 2.34 (0.12, 44)  p = 0.574 |
| Pitcherϑ | 2.14 (0.53, 8.70),  p = 0.287 | 0.61 (0.04, 8.70)  p = 0.717 |
| Professional* | 0.53 (0.18, 1.60),  p = 0.263 | 2.07 (0.26, 16.6)  p = 0.495 |
| Baseball Seasons | 1.01 (0.93, 1.10),  p = 0.843 | 1.03 (0.90, 1.18)  p = 0.637 |
| History of Joint InjuryΦ | 1.20 (0.30, 8.85),  p = 0.801 | 7.61 (0.74, 78)  p = 0.088 |
| Age | 1.04 (0.99, 1.08),  p = 0.080 | 1.07 (0.98, 1.16)  p = 0.117 |
| Body Mass Index | 0.96 (0.82, 1.13),  p = 0.652 | 0.95 (0.69, 1.31)  p = 0.743 |
| Right Handedψ | 0.96 (0.25, 3.70),  p = 0.958 | 0.04 (0.01, 0.76)  p = 0.032 |
| History of Orthopaedic SurgeryΘ | 0.41 (0.07, 2.44),  p = 0.325 | 1.56 (0.14, 17)  p = 0.716 |

95% CI = 95% Confidence Interval

Persistent pain was defined as pain on ‘*most days of the last most’*

ϑ Position players were used as the reference category in the multivariable analyses

* College baseball players were used as the reference category in the multivariable analyses

ψ Left handed players were used as the reference category in the multivariable analyses

Φ For the elbow persistent pain analyses, this is referring to a history of elbow joint injury, in contrast to the shoulder persistent pain analyses, this is referring to a history of shoulder joint injury

Θ For the elbow persistent pain analyses, this is referring to a history of elbow orthopaedic surgery, in contrast to the shoulder persistent pain analyses, this is referring to a history of a shoulder orthopaedic surgery

Appendix 8. Sensitivity Analysis: The adjusted odds and relationship of pitchers and non-pitchers and joint pain and arm function in former baseball players

| Variable | Persistent Shoulder/Elbow Pain | SANE |
| --- | --- | --- |
|  | Odd Ratios  (95% CI) | Beta  (95% CI) |
| Pitcherϑ | 4.36 (1.28, 11.9),  p = 0.019 | -2.29 (-15.67, 11.09),  p = 0.734 |
| Professional* | 1.74 (0.57, 5.29),  p = 0.323 | 1.47 (-11.64, 14.58),  p = 0.824 |
| Baseball Seasons | 1.03 (0.95, 1.11),  p = 0.471 | -0.09 (-1.02, 0.84),  p = 0.848 |
| History of Joint InjuryΦ | 0.97 (0.31, 2.98),  p = 0.951 | -4.47 (-17.90, 8.96),  p = 0.509 |
| Age | 1.01 (0.97, 1.05),  p = 0.620 | -0.16 (-0.62, 0.28),  p = 0.460 |
| Body Mass Index | 1.05 (0.90, 1.23),  p = 0.512 | -0.97 (-2.86, 0.93),  p = 0.312 |
| Right Handedψ | 0.31 (0.08, 1.12),  p = 0.073 | 0.92 (-13.88, 15.45),  p = 0.915 |
| History of Orthopaedic SurgeryΘ | 3.00 (1.04, 8.66),  p = 0.041 | -7.81 (-20.23, 4.60),  p = 0.213 |

SANE = Single Assessment Numeric Evaluation

95% CI = 95% Confidence Interval

Persistent pain was defined as pain on ‘*most days of the last most’*

ϑ Non pitchers (Two way and position players) were used as the reference category in the multivariable analyses

* College baseball players were used as the reference category in the multivariable analyses

ψ Left handed players were used as the reference category in the multivariable analyses

Φ For the persistent joint injury analyses, this is referring to a history of any joint injury, in contrast to the arm persistent pain and SANE analyses, this is referring to a history of a throwing arm joint injury

ΘFor the persistent joint injury analyses, this is referring to a history of any orthopaedic surgery, in contrast to the arm persistent pain and SANE analyses, this is referring to a history of a throwing arm orthopaedic surgery
